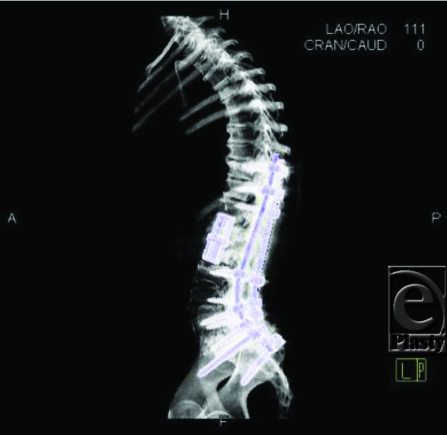# Reconstruction of Spinal Defect Using Free Fibular Transfer

**Published:** 2011-02-25

**Authors:** Allison E. Shen, Richard L. Agag, Steven Stavrides

**Affiliations:** Long Island Plastic Surgical Group, Garden City, NY

## DESCRIPTION

A 50-year-old man presents with a tumor of his thoracic spine. After resection by neurosurgery, there is a 10-cm defect, which requires reconstruction. The patient will receive postoperation radiation therapy.

## QUESTIONS

1. **What are the benefits of reconstructing a spinal defect?**
2. **What are the indications for a vascularized and nonvascularized bone graft?**
3. **What are the options for recipient blood vessels?**

## DISCUSSION

Spinal tumors are most often the result of metastasis of a preexisting tumor such as breast or prostate cancer. However, primary tumors may also arise in the spine; these may be either benign (eg, giant cell tumor, osteoblastoma) or malignant (eg, multiple myeloma, Ewing sarcoma). Whatever the cause, reconstruction of spinal defects is critical for stabilization of the spine. Although the hardware bears the majority of the stress during the first few months after reconstruction, as the bone graft incorporates, it is able to bear the majority of the stress. If the hardware needs to be removed at any point after this, the patient's spine will remain stabilized.

The type of bone graft used varies depending on the size of the defect and the need for radiation therapy. Nonvascularized grafts are indicated for small defects (<5 cm); however, these are not recommended in a radiated field because of higher failure rates. Nonvascularized bone grafts are more likely to fracture and may become increasingly porous over time.

Vascularized grafts are indicated for patients with larger defects (>5 cm) because they offer greater mechanical stability, incorporate more quickly, hypertrophy more rapidly, and are overall more effective at achieving bony union. They are also more resistant to infection in the postoperative period. Vascularized bone grafts are also more preferable for patients requiring radiation therapy, as they have been shown to have substantially greater bone density than nonvascularized grafts even several years postoperatively. The options for reconstruction with vascularized bone grafts include rib, iliac crest, and fibula depending on the size of the defect. Free fibula grafts offer the greatest length; they are able to reconstruct defects up to 26 cm.

The choice of recipient vessels for spinal reconstruction is limited. The cervical spine generally presents the fewest difficulties because of the number of recipient blood vessels in close proximity, such as the transverse cervical artery and branches of the external carotid artery. Reconstruction of the thoracic spine is more challenging, but can be accomplished through utilization of the internal thoracic vessels. Defects of the lumbar spine may require the use of the mesenteric or lumbar segmental vessels and the iliac vessels are available for use in reconstructing the sacrum or pelvis. In any of these locations, an arteriovenous loop may be necessary to achieve adequate pedicle length.

## Figures and Tables

**Figure:**
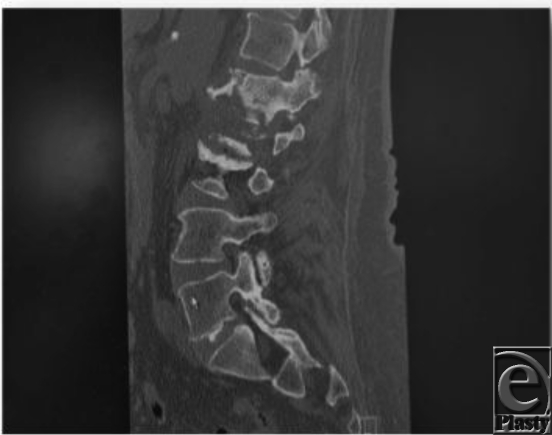


**Figure:**
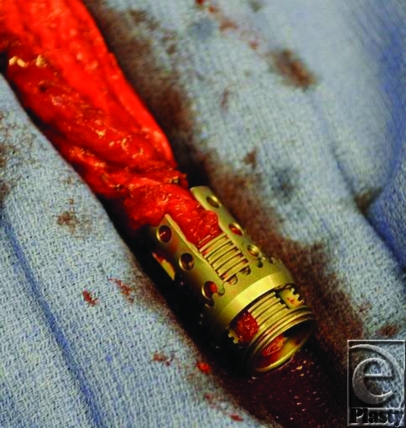


**Figure:**